# Supplementary material for: Identification of photoperiod-induced specific miRNAs in the adrenal glands of Sunite sheep (Ovis aries)
Source: Front Vet Sci. 2022 Jul 22;9:888207. doi: 10.3389/fvets.2022.888207 (PMC9354845; doi:10.3389/fvets.2022.888207)
Supplement: Supplementary file 1 [file Data_Sheet_1.ZIP › Supplementary file/miRDeep2 file.zip!/miRDeep2 file/Know_Structure/SP42A1/oar-mir-19b.pdf]

miRBase precursor : oar-mir-19b
Total read count : 2286
oar-miR-19b read count : 2279
remaining reads : 7

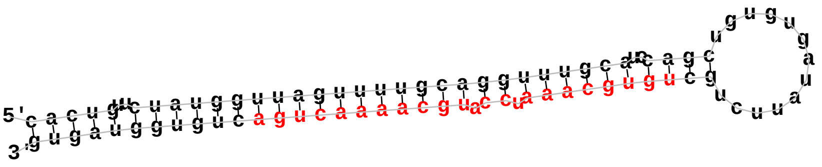

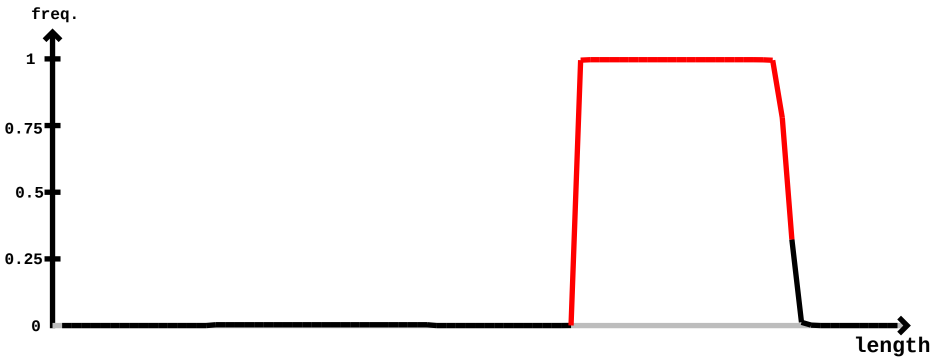

oar-miR-19b

```
5'- cacuguucuaugguuaguuuugcagguuugcauccagcugugugauauucugcugugcaaauccaugcaaaacugacugugguagug   -3'    exp
    (((((..((((((((((((((((((((((((((..((((............)))))))))).)).))))))))))))))))))))))   reads      mm         sample
    ...............aguuuugcagguuugcauccagc.................................................   7          0          seq
    ....................................................cugugcaaauccaugcaaaacuga...........   1          0          seq
    .....................................................ugugcaaauccaugcaaaa...............   1          0          seq
    .....................................................ugugcaaauccaugcaaaac..............   4          0          seq
    .....................................................ugugcaaaucUaugcaaaacu.............   55         1          seq
    .....................................................ugugcaaauccaugcaaaacu.............   438        0          seq
    .....................................................ugugcaaaucUaugcaaaacug............   76         1          seq
    .....................................................ugugcaaauccaugcaaaacug............   966        0          seq
    .....................................................ugugcaaauccaugcaaaacuga...........   612        0          seq
    .....................................................ugugcaaaucUaugcaaaacuga...........   95         1          seq
    .....................................................ugugcaaauccaugcaaaacugac..........   2          0          seq
    .....................................................ugugcaaauccaugcaaaacugaU..........   21         1          seq
    .....................................................ugugcaaauccaugcaaaacugaUu.........   2          1          seq
    .....................................................ugugcaaauccaugcaaaacugacu.........   2          0          seq
    ......................................................gugcaaauccaugcaaaacug............   2          0          seq
    ......................................................gugcaaaucUaugcaaaacuga...........   1          1          seq
    ......................................................gugcaaauccaugcaaaacugac..........   1          0          seq
```
